# Improving plant transient expression through the rational design of synthetic 5′ and 3′ untranslated regions

**DOI:** 10.1186/s13007-019-0494-9

**Published:** 2019-09-18

**Authors:** Hadrien Peyret, James K. M. Brown, George P. Lomonossoff

**Affiliations:** 1 Department of Biological Chemistry, John Innes Centre, Norwich Research Park, Norwich, NR4 7UH UK; 2 Department of Crop Genetics, John Innes Centre, Norwich Research Park, Norwich, NR4 7UH UK

**Keywords:** pHREAC, pEAQ, pHRE, Deconstructed vectors, Molecular farming, Plant transient expression, Recombinant protein, Viral expression system, UTR, Synthetic biology

## Abstract

**Background:**

The growing field of plant molecular farming relies on expression vectors that allow high yields of recombinant proteins to be produced through transient gene expression. While numerous expression vectors currently exist for this purpose, there are very few examples of systematic efforts to improve upon these. Moreover, the current generation of expression systems makes use of naturally-occurring regulatory elements, typically selected from plant viruses, to maximise yields. This study aims to use rational design to generate synthetic sequences that can rival existing ones.

**Results:**

In this work, we present the rational design of novel synthetic 5′ and 3′ untranslated regions (UTRs) which can be used in various combinations to modulate accumulation levels of transiently-expressed recombinant proteins. Using the pEAQ-*HT* expression vector as a point of comparison, we show that pre-existing expression systems can be improved by the deployment of rationally designed synthetic UTRs. Notably, we show that a suite of short, synthetic 5′UTRs behave as expression enhancers that outperform the *HT* 5′UTR present in the CPMV-*HT* expression system. Furthermore, we confirm the critical role played by the 3′UTR of cowpea mosaic virus RNA-2 in the performance of the CPMV-*HT* system. Finally, we use the knowledge obtained from these results to develop novel expression vectors (named pHRE and pHREAC) that equal or outperform pEAQ-*HT* in terms of recombinant protein yield. These new vectors are also domesticated for the use of certain Type IIS restriction enzymes, which allows for quicker cloning and straightforward assessment of different combinations of UTRs.

**Conclusions:**

We have shown that it is possible to rationally design a suite of expression modulators in the form of synthetic UTRs. We have created novel expression vectors that allow very high levels of recombinant protein expression in a transient expression context. This will have important consequences for future efforts to develop ever-better plant transient overexpression vectors for research or industrial applications.

## Background

The field of plant molecular farming has emerged as an exciting area of research at the intersection between plant biotechnology and industrial bioengineering [17]. Proteins, small molecules and biologics produced in plants are beginning to reach the market and plants are becoming a serious alternative to unicellular expression hosts for the production of recombinant proteins [10]. Underpinning these developments are transient expression systems [16]. However, most of the current expression systems have undergone limited (if any) systematic optimisation, and the viral regulatory components that they use have typically not been compared with potential, synthetic alternatives. Indeed, they typically make use of wild-type or minimally altered regulatory components from just one or two plant viruses and rely on the natural characteristics of these components to achieve high recombinant protein yield. Moreover, while many expression systems make use of 5′ and 3′ untranslated regions (UTRs) as enhancers of expression, there have, to our knowledge, been no systematic attempts to create synthetic UTRs de novo. It is generally assumed that viral UTRs are excellent natural translational enhancers, and yet in the evolutionary context of plant RNA viruses (those most often used to construct expression systems), the UTRs have multiple roles during the virus life cycle [20]. Importantly, two of these roles are somewhat contradictory: the UTRs must allow synthesis of negative-stranded RNA for viral RNA replication (which moves in the 3′ to 5′ direction along a positive-stranded viral RNA) as well as efficient translation of viral proteins (which moves in the 5′ to 3′ direction along the same positive-stranded RNA). This means that viral UTRs must strike a balance between different functions in order to optimise the viral replication cycle. In a recombinant plant expression system, however, the goal is typically maximal expression and accumulation of one or a few recombinant proteins of interest which, in non-replicating systems, requires only efficient translation.

In light of this, we wished to investigate the possibility of using a synthetic biology approach to develop a novel, efficient transient expression system. We have used the successful pEAQ-*HT* expression vector [19] (GenBank accession number GQ497234.1) as a starting point and comparator in an iterative process intended to test combinations of novel, rationally-designed synthetic UTRs. The pEAQ-*HT* vector is based on the CPMV-*HT* expression system which uses the UTRs of RNA-2 of cowpea mosaic virus (CPMV) to direct expression of a gene of interest placed between a 35S promoter and nos terminator [18]. Importantly, the 5′UTR is mutated (the *hypertrans* or *HT* mutations) such that upstream AUG codons are removed, which greatly enhances translational efficiency. By contrast, the contribution of the 3′UTR to final protein yield was unknown [15] until Meshcheriakova et al. [12] determined that it does, in fact, play a key role by increasing mRNA accumulation within cells. Thus, it behaves orthogonally to the 5′UTR, which increases translational efficiency but not mRNA levels [18]. This suggests that mixing and matching orthogonally acting UTRs might be a logical strategy for modulating protein expression in transient expression systems. In addition to the CPMV-based UTRs, pEAQ-*HT* includes an expression cassette for the P19 suppressor of gene silencing from tomato bushy stunt virus (TBSV) in the T-DNA alongside the *HT* expression cassette to increase mRNA stability [19]. Together these features make pEAQ-*HT* a popular choice for the transient expression of recombinant proteins in the host plant *Nicotiana benthamiana*. Indeed, this vector has been used by numerous labs around the world [15] due to its ease of use and high yield of recombinant protein: it is known to direct GFP expression at yields above 1.5 g per kg of fresh weight tissue [19].

The work presented here addresses the hypothesis that naturally occurring UTRs, or slight variations thereof, are not necessarily the best options for maximising recombinant protein yield in the context of plant transient expression systems. In essence, we sought to determine whether the tools that have evolved naturally are the best available. To answer this, we describe the creation of new expression vectors with pEAQ-*HT* as the starting point. Novel, synthetic 5′ and 3′UTRs were designed and tested in various combinations to examine if it is possible to create novel 5′ and 3′UTRs that can outperform those present in pEAQ-*HT*. Some of these synthetic UTRs were then deployed in the design and construction of novel expression vectors that have advantages in terms of ease of cloning and expression yield compared to pEAQ-*HT*.

## Results

### Creation of the Synth expression cassette

**Fig. 1:**
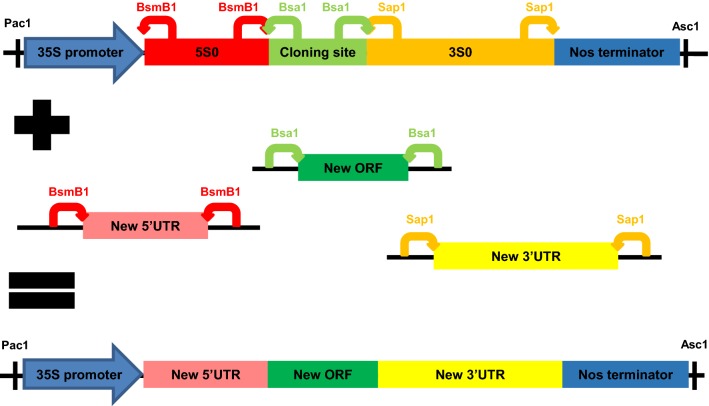
The Synth expression cassette. Diagram of the Synth expression cassette used in this study, with arrows indicating location and cut sites of the different Type IIS restriction sites. These allow easy replacement of the UTRs or cloning site with new UTRs and ORFs of interest in one-pot restriction/ligation reactions. The complete annotated sequence of Synth can be found in Additional file 1

**Fig. 2:**
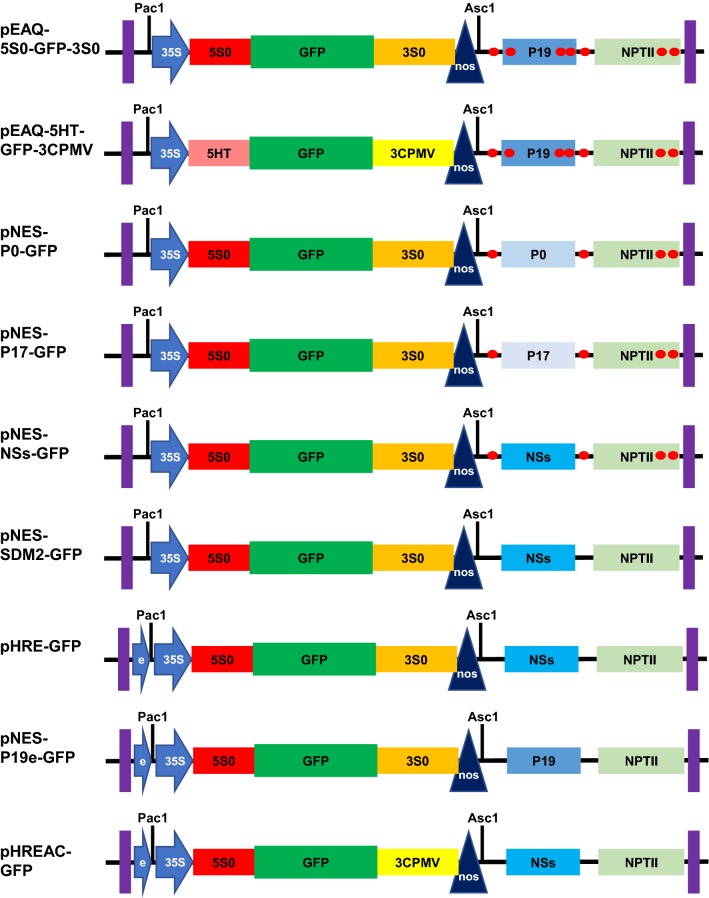
Diagrams of the key T-DNAs used in this study. Purple bars: left and right T-DNA borders. Blue arrows represent promoters, with “35S” representing the full-length 35S promoter, while “e” represents the 35S promoter enhancer region. Red boxes represent 5′UTRs, green boxes represent the GFP ORF, orange boxes represent 3′UTRs, dark blue triangles represent the *nos* terminator, blue rectangles represent silencing suppressor genes (which are under the control of the 35S promoter and terminator, not shown), and NPTII represents the *nptII* kanamycin resistance gene (not to scale). Red dots represent Bsa1, Sap1 and BsmB1 restriction sites, while Pac1 and Asc1 restriction sites are labelled directly. The vector backbones (not shown) are the same as in pEAQ-*HT* [19]

A novel expression cassette (named Synth) was designed to allow straightforward replacement and testing of different UTRs (see Fig. 1). This Synth cassette is based on the CPMV-*HT* expression cassette present in the pEAQ-*HT* vector: it contains the same 35S promoter and nos terminator sequences [19], but with synthetic 5′ and 3′UTRs replacing the CPMV-based UTRs present in CPMV-*HT*. These UTRs were named 5S0 at the 5′ end and 3S0 at the 3′ end, and their sequences are available in Additional file 1 (in the context of the Synth cassette) and Additional file 2 (individually). Each of these synthetic UTRs contains Type IIS restriction sites at either end (BsmB1 in the 5′UTR and Sap1 in the 3′UTR) which cut outwards, allowing the UTR to be easily replaced with an alternative sequence, as shown in Fig. 1. Between the two UTRs, a pair of Bsa1 sites was introduced to allow insertion of a coding sequence of interest between the UTRs via one-pot restriction/ligation. The new vectors generated thanks to Synth were named in such a way as to highlight the UTR combination used: for example, the pEAQ backbone carrying the Synth-GFP cassette into which the GFP ORF and the original Synth 5S0 5′UTR and 3S0 3′UTR were present was named pEAQ-5S0-GFP-3S0 (see Fig. 2). The end-tailored versions of the 5′ and 3′UTRs of the CPMV-*HT* expression cassette (5HT and 3CPMV, see Methods) were also inserted into Synth-GFP using the appropriate Type IIS restriction sites in order to create a version of pEAQ-*HT* that used the same scarless cloning strategy as the Synth-based vectors. This vector was named pEAQ-5HT-GFP-3CPMV (see Fig. 1 for the cloning strategy and Fig. 2 for diagrams of the key resulting T-DNAs).

### Comparisons of various combinations of UTRs

**Fig. 3:**
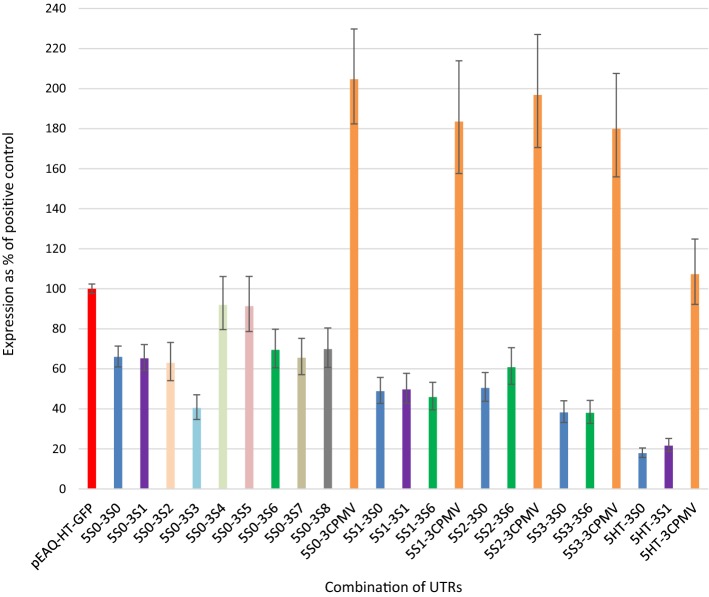
Relative GFP expression levels for different UTR combinations. GFP was expressed transiently from expression cassettes using different combinations of 5′ and 3′ UTRs, as indicated on the x-axis. GFP fluorescence was measured in soluble protein extract obtained from agroinfiltrated leaves, and expression is shown relative to pEAQ-*HT*-GFP (100%, red bar). Bars with the same colour represent constructs with the same 3′UTR. For each construct, n ≥ 5. Error bars represent 95% confidence intervals

Expression levels of the different UTR combinations were compared to one another and to pEAQ-*HT* by measuring GFP fluorescence in protein extracts obtained from agroinfiltrated *N. benthamiana* leaves. The results, shown in Fig. 3, reveal that 5S0 is the best 5′UTR of those tested, and constructs using 5S0 express approximately twice as much GFP as constructs using 5HT, the 5′UTR present in pEAQ-*HT* (all other things being equal). In fact, 5HT was found to be a worse 5′UTR (in terms of protein expression) than any of the synthetic 5′UTRs tested, in combination with each of the three different 3′UTRs with which it was tested. This was not the case for the 3′UTR, however. Indeed, 3CPMV, which is the 3′UTR present in pEAQ-*HT*, was found to give very significantly higher yield of GFP than any of the synthetic 3′UTRs tested, irrespective of the 5′UTR. This held true even when synthetic 3′UTRs contained the Y-loop structure from 3CPMV which is known to have a major role in overall protein expression [12], although the synthetic UTRs which contain the Y-loop (3S4, 5, 6, 7, and 8) did tend to outperform those without (3S0, 1, 2, and 3). However, modifying the sequence of the Y-loop in a way designed to preserve its structure (3S6, 7 and 8) led to lower GFP fluorescence than keeping the sequence intact (3S4 and 5). This was unexpected, as Meshcheriakova et al. [12] suggested that the yield-boosting effect of the CPMV Y-loop was a function of its structure and not its sequence. Attempts were made to create simple secondary structures that could allow base-pairing interactions between 5′ and 3′ UTRs (such as simplified 3′CITE and CIRE component loops; see Tables 1 and 2 and Additional file 2) but there was no evidence that any of these sequences caused synergistic effects between the 5′ and 3′ UTRs that contained the corresponding parts, such as 5S3 and 3S6. Instead, the different 5′ and 3′ UTRs consistently functioned orthogonally, with their effect on yield functioning in an additive manner rather than synergistically. This confirms previous findings by Meshcheriakova et al. [12], who determined that the *HT* 5′UTR and 3′UTR of the CPMV-*HT* cassette function independently.

**Table 1:** The properties of the 5′UTRs used in this study

5′UTR	UTR length (bp)	GC content (%)	AAC motifs	Free energy (kcal/mol)^a^	Deliberately designed structures
5S0	64	34	4	− 4.27	None
5S1	60	30	5	− 1.4	None
5S2	72	32	4	− 10.7	Synthetic CITE^b^ component loop
5S3	93	31	5	− 10.5	Synthetic CITE^b^ and CIRE^c^ component loops
5HT	512	41	1	− 110.84	None (viral sequence)

The properties of the 5′UTRs used in this study. ^a^ Measured by the mFold secondary structure prediction server [24]; ^b^ CITE: 3′Cap-independent translation enhancer; ^c^ CIRE: Cap-independent regulatory element

**Table 2:** The properties of the 3′UTRs used in this study

3′UTR	UTR length (bp)	GC content (%)	CA motifs	AAUAAA motifs	UUUU motifs	Free energy (kcal/mol)^a^	CPMV 3′UTR Y-loop	Deliberately designed structure
3S0	202	36	20	3	5	− 17.5	No	None
3S1	200	34	25	4	6	− 13.1	No	None
3S2	50	36	7	1	1	− 1	No	None
3S3	135	34	9	1	5	− 23.7	No	None
3S4	175	38	11	1	1	− 28.92	Wt	Synthetic CITE^b^
3S5	179	39	14	1	1	− 34.13	Wt	None
3S6	168	38	10	1	0	− 30.52	Totally divergent	Synthetic CITE^b^
3S7	177	42	9	1	0	− 35.07	Totally divergent	Synthetic CITE^b^ + BTE^c^
3S8	168	38	10	1	0	− 31.32	Partially divergent	Synthetic CITE^‡^
3CPMV	185	33	8	0	5	− 28.73	Wt	None (viral sequence)

The properties of the 3′UTRs used in this study. ^a^ Measured by the mFold secondary structure prediction server [24]; ^b^ CITE: 3′Cap-independent translation enhancer; ^c^ BTE: barley yellow dwarf virus-like translation element

### Comparing the effect of different suppressors of silencing

**Fig. 4:**
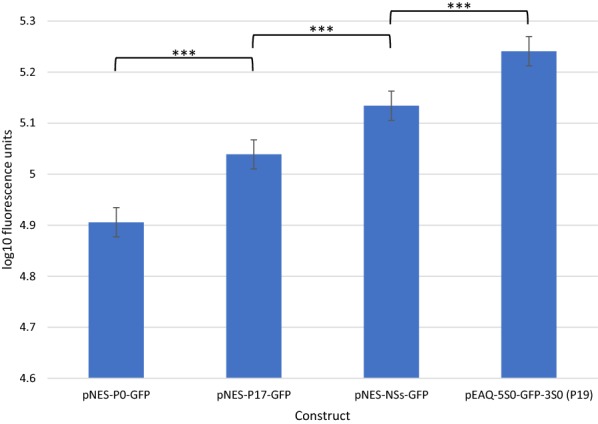
GFP expression levels for different silencing suppressors. GFP was expressed transiently from expression cassettes using the 5S0 and 3S0 UTRs but different viral silencing suppressors, as indicated on the x-axis. GFP fluorescence was measured in soluble protein extract obtained from agroinfiltrated leaves, and three biological replicates (n = 3) for each construct were all measured on the same 96-well plate. Error bars represent 95% confidence intervals around the predicted mean; *** represents p < 0.001

Plasmid pEAQ-5S0-GFP-3S0 was modified to allow replacement of P19 to ascertain the impact of different silencing suppressors on GFP expression. Alternative plant viral silencing suppressors were chosen that belong to well-characterised classes of silencing suppressors, but which (to our knowledge), have not yet been used in transient expression systems, and could therefore represent an untapped resource. These were P0 from tobacco vein distorting virus (Polerovirus), P17 from cucumber leaf spot virus (Aureusvirus), and NSs from tomato zonate spot virus (Tospovirus). The coding sequence for each of these was used to replace the coding sequence of P19 (under the control of the 35S promoter and terminator) in pEAQ-5S0-GFP-3S0. The resulting plasmids contain neither the P19 silencing suppressor nor the CPMV-*HT* expression cassette that characterise the pEAQ suite of vectors [15], but instead constitute novel expression systems, and so these new plasmids were named pNES-P0-GFP, pNES-P17-GFP, and pNES-NSs-GFP, respectively (see Fig. 2). These were tested alongside pEAQ-5S0-GFP-3S0 in triplicate infiltration tests, which revealed that none of these silencing suppressors support GFP expression as well as P19, with NSs coming closest (see Fig. 4).

### Using rational design and novel UTRs to create novel expression vectors

**Fig. 5:**
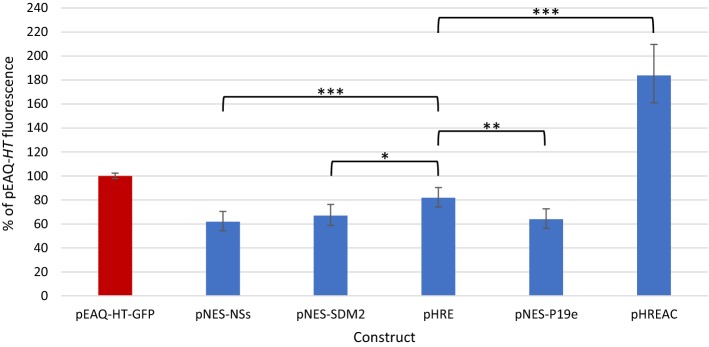
Relative GFP expression levels for different backbone and UTR combination. GFP was expressed transiently from expression cassettes using different combinations of 5′ and 3′ UTRs and different backbones (see Fig. 2), as indicated on the x-axis. GFP fluorescence was measured in soluble protein extract obtained from agroinfiltrated leaves, and expression is shown relative to pEAQ-*HT*-GFP (100%, red bar). For each construct, n ≥ 6. Error bars represent 95% confidence intervals; * represents p < 0.05; ** represents p < 0.01; *** represents p < 0.001

To maximise protein expression and simplify future cloning, a series of modifications were made to pNES-NSs-GFP to improve elements outside of the expression cassette. The plasmid was domesticated for the use of Type IIS restriction sites by site-directed mutagenesis of the Bsa1, BsmB1, and Sap1 restriction sites present both in the T-DNA (shown by red dots in Fig. 2) and in the vector backbone: in the *trfA* locus and the *colE1* origin of replication sequence [19]. The EcoR1 sequence just upstream of the 35S promoter controlling the expression of the silencing suppressor was also removed to make future replacement of the silencing suppressor more straightforward. This domesticated version of pNES-NSs-GFP was named pNES-SDM2-GFP (Fig. 2). In an effort to boost expression as much as possible, pNES-SDM2-GFP was further modified by the introduction of the 35S enhancer region of the 35S promoter [5, 7] just upstream of the 35S promoter that controls the expression of GFP. This domesticated plasmid, which makes use of the duplicated 35S promoter strategy, was expected to direct high recombinant expression and was therefore named pHRE-GFP (see Fig. 2). Expression tests revealed that site-directed mutagenesis did not adversely affect yield, and the addition of the 35S enhancer sequence increased the accumulation of GFP to a modest but statistically significant degree (p = 0.02, see Fig. 5). Because NSs had originally been determined to be less effective than P19 at boosting recombinant protein yield (see Fig. 4), the sequence of P19 was domesticated to remove BsmB1 and Bsa1 sites and cloned into pHRE-GFP to replace NSs. Surprisingly, this plasmid, which was named pNES-P19e-GFP (see Fig. 2), did not perform as well as pHRE-GFP (p = 0.002, Fig. 5). This may suggest that use of a duplicated 35S promoter increases recombinant protein yield in a vector that uses the NSs silencing suppressor but not in a vector using the P19 silencing suppressor. However, it is also possible that the silent mutations introduced into the P19 gene had a negative impact on its expression. In any case, pHRE-GFP was the best domesticated vector with the best 5′UTR controlling expression of GFP. In an attempt to make an even better vector, the 3CPMV 3′UTR was cloned into pHRE-GFP (where it replaced 3S0) to yield a plasmid named pHREAC-GFP (for high recombinant expression associated with CPMV, see Fig. 2). This vector significantly outperformed both pHRE-GFP and pEAQ-*HT*-GFP (p < 0.001 in both cases), with nearly double the fluorescence generated by the latter (Fig. 5). Based on previously published yield of GFP obtained with pEAQ-*HT* [19], we can estimate that pHREAC-GFP produces about 3 g of GFP per kg of fresh weight tissue.

**Fig. 6:**
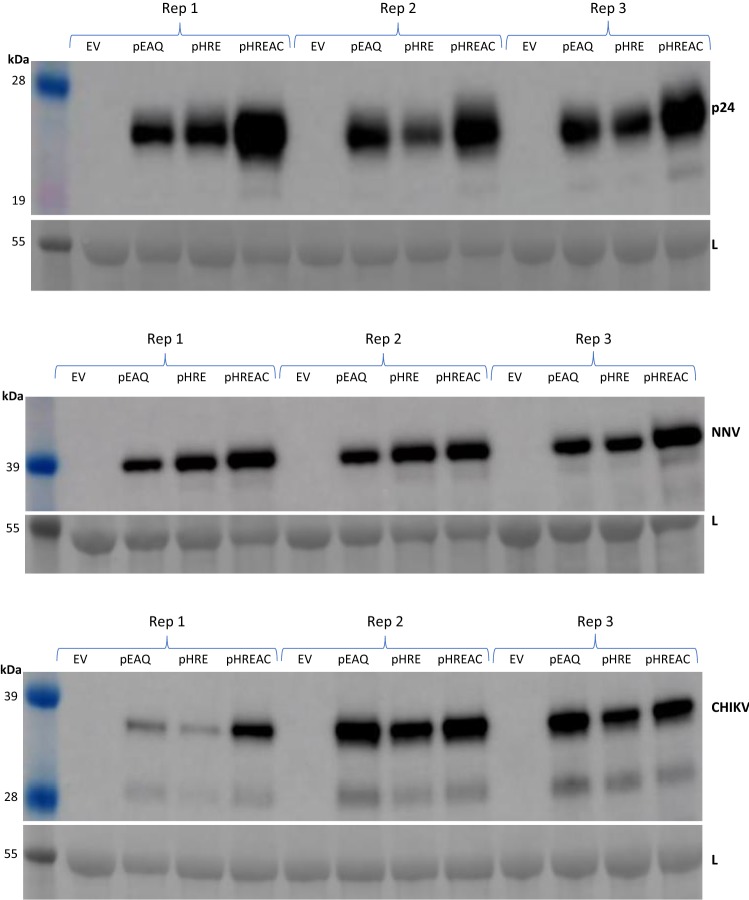
Western blots assaying expression tests of three different proteins. The pEAQ, pHRE, and pHREAC vectors were used to transiently express three different proteins: the p24 protein of HIV (top), the coat protein of NNV (middle) and the capsid protein of chikungunya virus (bottom). Soluble protein extracts from three biological replicates (Rep 1,2, and 3) were electrophoresed on the same SDS-PAGE gel along with extract from empty vector (EV)—infiltrated leaves, then transferred to nitrocellulose membrane for protein-specific western blotting. After chemiluminescent detection, each membrane was stained with Ponceau Red to reveal protein loading based on the large subunit of RuBisCO (shown below each blot)

The work described so far used GFP as the protein of interest. To test whether the relative expression efficiencies with these vectors hold true for proteins other than GFP, “empty vector” (EV) versions of pHRE-GFP and pHREAC-GFP which contain only the Bsa1 cloning site from Synth between the UTRs were generated and named pHRE-EV and pHREAC-EV, respectively. These were then used for the cloning and expression of three different coding sequences: the p24 sequence from HIV, the coat protein (CP) sequence from nervous necrosis virus (NNV), and the entire structural open reading frame (ORF2) from chikungunya virus. These were chosen because there are no Bsa1, BsmB1 or Sap1 sites in these sequences, and pEAQ-*HT* vectors containing these constructs were already available in the laboratory. The expression of these proteins could therefore be compared in the context of the three different vectors: pEAQ-*HT*, pHRE, and pHREAC. The expression comparison experiments were carried out on three separate occasions and the recombinant proteins were identified in soluble protein extract from agroinfiltrated leaves by western blot (Fig. 6). This showed that proteins accumulate to similar levels when expressed with pHRE or pEAQ-*HT*, but expression with pHREAC is generally higher, although this was less clear for the chikungunya virus construct. This demonstrates that the expression results obtained with GFP largely hold true for three completely different sequences expressed with these three vectors.

## Discussion

The work presented here had two goals. The first was to investigate the use of synthetic UTRs in plant transient expression systems, to determine whether they could surpass naturally occurring UTRs in terms of recombinant protein yield. The second was to use rational design to create a new overexpression vector, with the best UTRs and the optimal vector backbone, which could maximise protein expression levels. Not only is this work relevant in terms of biotechnology, but it also offers valuable insight into the role of UTRs for translation in plants. Indeed, the results presented here show that generating synthetic 5′UTRs that behave as strong translational enhancers is straightforward. Relatively short sequences with low GC content, multiple CA motifs and low secondary structure favour efficient translation. However, the best 5′UTR (5S0) was not the one with the most CA motifs, nor was it the one with the lowest secondary structure (see Table 1). It is possible that there is a trade-off between RNA stability, which requires some secondary structure to avoid binding to regulatory short RNAs, and translational efficiency, which requires low secondary structure for optimal ribosomal binding. Crucially, all synthetic 5′UTRs tested were superior to the 5HT “hypertranslatable” modified 5′UTR from CPMV RNA-2 [18], regardless of the 3′UTR. By contrast, generating synthetic 3′UTRs that boost recombinant protein yield proved much more difficult. Indeed, none of the synthetic 3′UTRs that were generated were as successful as 3CPMV, the wild-type 3′UTR from CPMV RNA-2. Even using the Y-loop structure from 3CPMV [12] as a component of a synthetic 3′UTR did not generate the same protein accumulation as the original 3CPMV. Furthermore, none of the deliberately designed structures inspired by existing viral UTRs (CITE, CIRE, or BTE) had a clear effect on overall protein yield compared to UTR combinations which lacked these. In hindsight this is perhaps not surprising, since these deliberately designed structures are entirely removed from their natural UTR context. It is not clear why 3CPMV is so much better than the other 3′UTRs, except perhaps the lack of an AAUAAA motif, which has been described as a key polyadenylation signal, at least in mammalian cells [11]. However, it is worth noting that all 3′UTRs used in this study, including 3CPMV, were followed by the *nos* terminator, which does contain an AAUAAA motif, so in reality this motif was present in all 3′UTRs. Moreover, the *nos* terminator has recently been shown to be a suboptimal terminator to use for transient protein overexpression when compared to certain other naturally-occurring terminators and chimeric combinations thereof [2]. Therefore it may be of use to test these synthetic UTRs in combination with other terminator sequences to determine if their relative effects in terms of final protein yield still hold true.

Overall, it seems likely that what makes an efficient 3′UTR for translation is a combination of factors that cannot be reduced to simple sequence motifs or simple secondary structure elements. It is possible that secondary structure in the 3′UTR is important for protein accumulation in ways that are not yet clear: it is likely that this is due to a combination of RNA stability and transcriptional efficiency, and the trade-offs involved are very difficult to predict using the methods used here. In any case, the suite of synthetic 5′ and 3′UTRs that has been generated during the course of this work (the sequences of which can be found in Additional file 2) can now be used as orthogonally-functioning expression modulators. This is likely to be of great use to the plant biotechnology community, particularly for the co-expression of multiple proteins, as this often requires fine-tuning of the relative expression levels of the different proteins in order to maximise overall yield [21]. Moreover, this work demonstrates that creating synthetic UTRs and testing them in various combinations is relatively straightforward, which suggests that it might become a standard practice in the coming years, particularly for researchers with access to large gene expression datasets and software that can predict optimal synthetic UTR sequences (which were unavailable for this study).

The high levels of protein expression generated with CPMV-*HT* was initially ascribed solely to highly efficient “hypertranslation” associated with the 5′UTR [18], with the role, if any, of the 3′ UTR being unclear [15, 18]. However Meshcheriakova et al. [12] found that it also plays a key role in enhancing the levels of mRNA accumulation, a feature which operates in concert with (but not synergistically with) the translational enhancement provided by the *HT* 5′UTR. The work presented here goes further and suggests that the CPMV 3′UTR is a critical component of the high recombinant protein yield achievable with the CPMV-*HT* system, with the natural CPMV 5′UTR actually being a poor leader sequence that is de-repressed by the mutation of upstream AUG codons located within the 5′UTR (which result in the *HT* 5′UTR). Recent work on the link between replication and packaging in CPMV suggests that this phenomenon probably relates to balancing replication and translation of the viral RNA in the context of the viral life cycle [9]. This emphasises the fact that naturally-occurring sequences frequently used in overexpression vectors are not necessarily optimally suited to the task for which they are deployed, but rather carry over trade-offs from their role in nature.

## Conclusions

In addition to a collection of UTRs that can be used as expression modulators, the work reported here also resulted in the creation of two new expression vectors. The first, pHRE, gives similar expression levels to pEAQ-*HT*, and contains synthetic UTRs 5S0 and 3S0 which can easily be swapped out and replaced. This vector is ideal for rapidly and easily testing new combinations of UTRs, provided that they are flanked by the appropriate restriction sites (as shown in Fig. 1). The second vector, pHREAC, contains a synthetic 5′UTR (5S0) and the 3′UTR of CPMV, a combination that gave maximum recombinant protein yield. Both vectors allow easy, scar-less insertion of an open reading frame in a one-pot restriction/ligation reaction thanks to the unique Bsa1 restriction sites present between the UTRs. The sequences of these vectors have been deposited in GenBank (see accession numbers below). It is probable that both of these vectors will facilitate innovation in the field of plant molecular farming in the future.

This work may also have important implications for the deployment of plant transient expression systems beyond their current reach. The IP landscape surrounding plant molecular farming can, in some circumstances, create barriers to use of the most important tools [6]. This can discourage certain players (particularly from resource-poor and small-scale commercial backgrounds) from taking part fully in plant molecular farming, and overall this is detrimental to innovation. For this reason, an effort is being made to create novel tools that can be used by academic or commercial entities with as much freedom as possible thanks to novel intellectual property agreements and protocols such as the Open Material Transfer Agreement (OpenMTA) [6].

## Methods

### Design of the Synth expression cassette

When designing the Synth cassette, care was taken with the restriction site overhangs to ensure that cloning of UTRs and ORFs is directional, and that the 5′UTR ends with a favourable Kozak context. New UTRs designed during this work (summarised in Tables 1 and 2 and with sequences available in Additional file 2) contained the appropriate restriction sites and overhangs at the ends cutting inwards, in order to easily replace 5S0 and 3S0 with a new UTR, eliminating the restriction sites in the process. The Synth expression cassette was synthesized by GeneArt (Life Technologies) and the plasmid that it was delivered in (named EXP) was used as an entry clone for the cloning of each new combination of 5′ and 3′ UTRs using the Type IIS enzymes before the whole cassette was transferred to the pEAQ-*HT* backbone via Pac1/Asc1 restriction/ligation for transient expression in *N. benthamiana*.

The sequence for GFP was initially used as the test ORF to compare the impact of different UTRs. The GFP coding sequence was inserted into the Synth cassette via the Bsa1 sites, and this GFP-containing version of Synth (named Synth-GFP) could be used to easily investigate the effect of different UTR combinations by swapping the 5′ and/or 3′UTRs thanks to the BsmB1 and Sap1 restriction sites (see Fig. 1). The sequence for GFP used in this study (available in Additional file 3) is a solubility-enhanced version of eGFP [1, 14] which does not contain BsmB1, Bsa1 or Sap1 restriction sites, which means it was compatible with the cloning strategy of the Synth cassette. For comparability, the pEAQ-*HT*-GFP plasmid used as a control for the experiments described here contains this same solubility-enhanced version of eGFP. All cloned plasmids were transformed into *E. coli* TOP10 (Life Technologies) and the sequences verified by sequencing before transforming into *Agrobacterium tumefaciens* LBA4404.

### Design of synthetic 5′UTRs

Four synthetic 5′UTRs were generated (see Table 1 for a summary and Additional file 2 for sequences). These were designed based on the information available in the scientific literature about the properties of 5′UTRs associated with highly expressed genes and used as translational enhancers in transient expression systems [3, 4, 8, 13, 22]. Overall, desirable characteristics appeared to be relatively short sequences (about 60–70 bp) with low GC content, low secondary structure, repeats of an AAC motif, and a strong Kozak consensus sequence (UUAAAA immediately preceding the AUG start codon—the Kozak sequence elements downstream of the AUG are dependent on the protein coding sequence and therefore cannot be designed into a general-purpose expression system). Furthermore, intron start sites and upstream AUG start codons were completely avoided. The first synthetic 5′UTR, named 5S0, was introduced into the Synth expression cassette at the design stage with BsmB1 restriction sites cutting outwards (see above and Fig. 1). The other 5′UTRs, named 5S1, 5S2 and 5S3, were designed with BsmB1 sites on the ends cutting inwards, such that one-step restriction-ligation could be carried out to replace 5S0 with any of the other 5′UTRs. Many viruses use 3′Cap-Independent Translation Enhancer (3′CITE), which are sequences exposed on the surface of loops in the 3′UTR that base-pair with complementary sequences exposed on loops in the 5′UTR [13]. A simplified synthetic 3′CITE was created in some of the synthetic 3′UTRs (see below and Table 2) and the sequence that it base-pairs with is present in all four synthetic 5′UTRs, although only 5S2 and 5S3 have that sequence exposed on the surface of a loop, which increases their overall predicted secondary structure compared to 5S0 and 5S1 (see Table 1). This loop on 5S3 was designed to also include a side-loop which constitutes a simplified synthetic component of a cap-independent regulatory element (CIRE): a 7-nucleotide sequence with complementarity to a region of 18S rRNA. The original CIRE is known to improve translational efficiency in tobacco etch virus (TEV, [23]).

The synthetic UTR sequences were analysed by the mFold secondary structure prediction server [24] in order to ensure that structural elements were well-designed. The 5S0 UTR in the Synth-GFP cassette was replaced with the other 5′UTRs through BsmB1 restriction-ligation, followed by sub-cloning the expression cassette into the pEAQ backbone. As a control, the *HT* 5′UTR present in the CPMV-*HT* expression cassette [18] was end-tailored to flank it with BsmB1 restriction sites so that it too could be deployed in Synth-GFP in the same way as the synthetic 5′UTRs. This version of the *HT* UTR was named 5HT.

### Design of synthetic 3′UTRs

Nine synthetic 3′UTRs were generated (see Table 2 for a summary and Additional file 2 for sequences). The first, 3S0, was introduced into the Synth expression cassette at the design stage with Sap1 restriction sites cutting outwards (see Fig. 1). The other 3′UTRs, 3S1, 3S2 and 3S3, 3S4, 3S5, 3S6, 3S7 and 3S8, along with 3CPMV (see Table 2) were designed with Sap1 sites on the ends cutting inwards, such that one-step restriction-ligation could be carried out to replace 3S0 with any of the other 3′UTRs. The synthetic UTRs were designed based on the information available in the scientific literature about the properties of 3′UTRs associated with highly expressed genes and plant viruses reaching high titres in infected plants [4, 11, 13, 22]. Overall, desirable characteristics seemed to be low GC content, presence of CA sequences to signal polyadenylation, with AAUAAA sequences upstream and potentially UUUU sequences downstream of the CA sequences. Naturally-occurring 3′UTRs vary greatly in length, so 170–200 bp was chosen for most of the synthetic 3′UTRs because it is easier to control the different structural elements of the UTR with a shorter sequence.

As with 5′UTRs, intron start and end sites were avoided. The 3S1 3′UTR was made to be extremely similar to 3S0, but with the Sap1 sites on the outside cutting inwards such that they would be removed upon cloning into Synth-GFP. 3S2 is much shorter (50 bp) than the others to test the effect of size. 3S3 has a similar distribution of CA, AAUAAA, and UUUU motifs as 3CPMV, but without the 3CPMV secondary structure. 3S4 and 3S5 both contains the Y-loop structure of 3CPMV which is known to have an effect on final protein yield [12], and 3S4 also contains a simplified synthetic 3′CITE consisting of a small stem loop with a sequence complementary to a section of the synthetic 5′UTRs. 3S6 is very similar to 3S4 but the Y-loop sequence has been changed in such a way as to preserve its secondary structure while making the sequence as divergent as possible. 3S7 is similar to 3S6 but also contains an extra stem loop in the 3′CITE that corresponds to the 17-nucleotide conserved region of the barley yellow dwarf virus-like translation element (BTE): ggatcctgggaaacagg [13]. 3S8 is very similar to 3S6 but the sequence of the Y-loop is modified to be slightly more similar to wildtype (as it is in 3S4 and 3CPMV) as opposed to totally divergent. These characteristics are summarised in Table 2 and the sequences of these 3′UTRs are available in Additional file 2.

All UTR sequences were analysed by the mFold secondary structure prediction server [24] to ensure that the structural elements were well designed. The 3S0 UTR in Synth-GFP was replaced with the other 3′UTRs through Sap1 restriction-ligation, before sub-cloning the expression cassette into the pEAQ backbone for expression testing. As a control, the 3′UTR present in the CPMV-*HT* expression cassette [18] was end-tailored to flank it with Sap1 restriction sites so that it could be deployed in Synth-GFP in the same way as the synthetic 3′UTRs. This version of the 3′UTR was named 3CPMV.

Apart from 5S0 and 3S0, all of the novel synthetic 5′ and 3′ UTRs were generated using overlapping extension PCR to produce the sequences shown in Additional file 2. The resulting PCR products were then used in BsmB1 or Sap1 restriction-ligation (for 5′ and 3′ UTRs, respectively) for replacement of 5S0 or 3S0 in the Synth-GFP expression cassette.

### Backbone modification

Elements of the pEAQ-*HT* plasmid outside of the expression cassette were modified to achieve the desired outcomes. Different versions of the EcoR1 restriction fragment of pEAQ-*HT* were ordered for synthesis from GeneArt with the open reading frame (ORF) of P19 replaced with that of other viral suppressors of gene silencing. These were: the P17 protein of cucumber leaf spot virus (Aureusvirus, GenBank: DQ227315.1), the NSs protein of tomato zonate spot virus (Tospovirus GenBank: NC_010489.1), and the P0 protein of tobacco vein distorting virus (Polerovirus, GenBank: NC_010732.1). All were optimised for *Nicotiana benthamiana* codon usage and cloned into pEAQ-5S0-GFP-3S0 using the EcoR1 restriction sites. Because these plasmids do not contain the same expression cassette as pEAQ-*HT* or the same silencing suppressor, they were deemed to be Novel Expression Systems and so renamed as the pNES set of vectors as shown in Fig. 2. The highest-expressing of these new vectors, pNES-NSs, was used for backbone domestication to remove all BsmB1, Bsa1 and Sap1 sites outside of the expression cassette, along with the EcoR1 site just downstream of the expression cassette. This was done through site-directed mutagenesis using the GeneArt SDM kit (Life Technologies). The resulting plasmid was named pNES-SDM2. This plasmid was then further modified using InFusion cloning (Takara-Clontech) to add the 35Se enhancer sequence of the 35S promoter [5] into the Pac1 site just upstream of the expression cassette 35S promoter. This effectively generates a duplicated 35S promoter strategy in the expression cassette [7], and this plasmid was named pHRE (High Recombinant Expression). The 3CPMV 3′UTR was then cloned into pHRE using Sap1 restriction-ligation, and the resulting plasmid was named pHREAC (High Recombinant Expression Associated with CPMV), as shown in Fig. 2.

### GFP expression tests

Every construct expressing GFP was tested for expression levels via fluorescence quantification. Overnight cultures of *Agrobacterium* carrying the different constructs were pelleted and resuspended in infiltration buffer (10 mM MES pH 5.6, 10 mM MgCl_2_, 0.1 mM acetosyringone) to an optical density of 0.4. The bacterial suspensions were then agroinfiltrated into leaves of *Nicotiana benthamiana* plants using a needle-less syringe. Seven days post-infiltration (dpi), agroinfiltrated leaves were harvested and a cork borer was used to take one leaf disc from the tips of three leaves per plant for two plants per construct (for a total of six leaf discs per construct). This procedure, from the bacterial culture to the leaf disc sampling, constituted one biological replicate. The 7 dpi time point was selected because a time course carried out with four of the constructs described (pEAQ-*HT*-GFP; pEAQ-5S0-GFP-3S0; pEAQ-5S1-GFP-3S0; pEAQ-5HT-GFP-3S0) showed that peak GFP fluorescence was consistently reached at 7 dpi, so this was used for all constructs for consistency. Leaf discs were lysed in a BeadRuptor with a ceramic bead and 270 µl of 100 mM sodium phosphate pH 7.2 supplemented with protease inhibitor (Roche Complete). The crude lysates were clarified by two successive rounds of centrifugation in a microcentrifuge at 8 °C for 10 min per spin. The soluble protein extract was then loaded in technical triplicates of 60 µl in a black 96-well plate, and the fluorescence was measured on a ClarioStar plate reader. Each 96-well plate contained biological replicates of pEAQ-*HT*-GFP and pEAQ-*HT*-EV (empty vector) plus three to ten other constructs. There were usually three biological replicates of a construct on a plate but occasionally fewer. All constructs shown in Figs. 3 and 5 were tested in two or more independent plates and in at least five biological replicates in total. There were as many side-by-side comparisons of different constructs on the same plate as practical to maximise the statistical power of the plate-to-plate comparison. For the silencing suppressor experiment shown in Fig. 4, three biological replicates of each construct in technical triplicates were analysed on the same 96-well plate.

### Statistical analysis

Data on fluorescence (arbitrary units) were analysed by linear mixed modelling. Fluorescence scores were log-transformed so the residuals had an approximately normal distribution and were independent of fitted values. For the data displayed in Figs. 3 and 5, the fixed effects were Plate + Construct and the random effects were the Biological Replicate of each Construct within a Plate and Technical Replicates within each Biological Replicate (residual term). Fluorescence data for the negative control, pEAQ-*HT*-EV, were omitted from the model so as not to distort comparisons between GFP-expressing constructs (fluorescence values for this control were always multiple orders of magnitude lower than those measured for GFP-expressing constructs). The overall effect of Construct was very highly significant (*F *= 59.89 with 28 and 175 degrees of freedom (df); *P *≪ 0.001). For the data in Fig. 4, which were obtained from one plate, the Plate term was omitted from the model above and all constructs were tested in three biological replicates. The effect of Construct was again very highly significant (*F *= 408.81; 4 and 30 df; *P *≪ 0.001). Confidence intervals were calculated from the standard errors of the predicted means following the mixed model analysis. Statistical analysis was done with Genstat v.18.

### Analysis of other proteins

The 3CPMV 3′UTR was cloned into the Synth expression cassette of the EXP plasmid by Sap1 restriction/ligation. Pac1 and Asc1 were then used to transfer this 3CPMV-containing Synth cassette as well as the original Synth cassette into pHRE in order to create (respectively) pHREAC-EV (GenBank: MK521430) and pHRE-EV (GenBank: MK521429). These empty vectors contain unique Bsa1 sites between the UTRs for easy insertion of an ORF of interest. These Bsa1 sites were used to clone three other coding sequences: the 672 bp sequence coding for the p24 protein of human immunodeficiency virus (HIV, GenBank: 2XT1_A), the 1014 bp sequence coding for the coat protein (CP) of nervous necrosis virus (NNV, GenBank: ABU95413.1), and the 3744 bp structural polyprotein ORF of chikungunya virus (GenBank: KJ451624). The resulting plasmids were named pHRE-p24, pHRE-NNV, pHRE-CHIK, pHREAC-p24, pHREAC-NNV, and pHREAC-CHIK. These constructs were tested for expression in *N. benthamiana* plants in three biological replicates as described above with pEAQ-*HT*—expressed proteins and pEAQ-EV (empty vector) as controls. Soluble protein extract was then assayed for presence of protein by immunoblot using an anti-p24 antibody (Abcam ab20365), an anti-NNV CP antibody (Abcam ab26812), or an anti-chikungunya virus coat protein antibody (IBT Bioservices 04-0008). After chemiluminescent detection, the western blot membranes were stained with Ponceau Red and destained briefly with water in order to assess equal loading of samples based on the presence of the large subunit of RuBisCO.

## Supplementary information

**Additional file 1.** Sequence of the Synth expression cassette. The different elements follow the same colour scheme as in Fig. 1: blue for the 35S promoter and *nos* terminator, red for the 5S0 5′UTR, green for the cloning site, and yellow for the 3S0 3′UTR. Restriction sites indicated in Fig. 1 are underlined in the sequence: Pac1 at the 5′ end, BsmB1 in 5S0, Bsa1 in the cloning site, Sap1 in 3S0, and Asc1 at the 3′ end.

**Additional file 2.** Sequences of the cloning fragments of the synthetic 5′ and 3′UTRs. Restriction sites (BsmB1 for 5′UTRs and Sap1 for 3′UTRs) are in bold and italicized. The sequence of the UTR once cloned into the expression cassette is underlined. Yellow: simplified synthetic CITE component complementary sequences. Green: synthetic CIRE component sequence. Blue: simplified BTE sequence. Magenta: CPMV RNA-2 3′UTR Y-loop structure.

**Additional file 3.** Sequence of GFP used in this study. This is a version of eGFP which contains solubility-enhancing point mutations [1, 14].

## Data Availability

The datasets used and/or analysed during the current study are available from the corresponding author on reasonable request. The following sequences can be publicly accessed on the GenBank repository using the accession numbers shown: pHRE-EV: GenBank MK521429. pHREAC-EV: GenBank MK521430. pEAQ-*HT*: GenBank GQ497234.1. P17 protein of cucumber leaf spot virus: GenBank DQ227315.1. NSs protein of tomato zonate spot virus: GenBank NC_010489.1. P0 protein of tobacco vein distorting virus: GenBank NC_010732.1. p24 protein of human immunodeficiency virus: GenBank 2XT1_A. Coat protein (CP) of nervous necrosis virus: GenBank ABU95413.1. Structural polyprotein ORF of chikungunya virus: GenBank KJ451624.
