# Supplementary material for: Improving plant transient expression through the rational design of synthetic 5′ and 3′ untranslated regions
Source: Plant Methods. 2019 Sep 18;15:108. doi: 10.1186/s13007-019-0494-9 (PMC6749642; doi:10.1186/s13007-019-0494-9)
Supplement: Supplementary file 1 — Additional file 1. Sequence of the Synth expression cassette. The different elements follow the same colour scheme as in Fig. 1: blue for the 35S promoter and nos terminator, red for the 5S0 5′UTR, green for the cloning site, and yellow for the 3S0 3′UTR. Restriction sites indicated in Fig. 1 are underlined in the sequence: Pac1 at the 5′ end, BsmB1 in 5S0, Bsa1 in the cloning site, Sap1 in 3S0, and Asc1 at the 3′ end. [file 13007_2019_494_MOESM1_ESM.docx]

**Ttaattaa**ggaaacctcctcggattccattgcccagctatctgtcactttattgagAagatagtggaaaaggaaggtggctcctacaaatgccatcattgcgataaaggaaaggccatcgttgaagatgcctctgccgacagtggtcccaaagatggacccccacccacgaggagcatcgtggaaaaagaagacgttccaaccacgtcttcaaagcaagtggattgatgtgatatctccactgacgtaagggatgacgcacaatcccactatccttcgcaagacccttcctctatataaggaagttcatttcatttggagaggtttaa**gagacg**caaccacaacgctctaacgcaatcaatctacattatattaaa**cgtctc**taaaaa**gagacc**taggtaactgactag**ggtctc**tcgcc**gaagagc**gcatcggatctaataataaacttaggcaataaatttcgacatcataataaagccacgtatctacatcaacacaagattagtattttcaaaactgttttcagcagaacacatattttcatttttaacgtaattttcatttagcgttagtacagtcaccttcacagaacgtttggagaagtgagg**gctcttc**cgatcgttcaaacatttggcaataaagtttcttaagattgaatcctgttgccggtcttgcgatgattatcatataatttctgttgaattacgttaagcatgtaataattaacatgtaatgcatgacgttatttatgagatgggtttttatgattagagtcccgcaattatacatttaatacgcgatagaaaacaaaatatagcgcgcaaactaggataaattatcgcgcgcggtgtcatctatgttactagatc**ggcgcgcc**

**Additional File 1 (.docx).** Sequence of the Synth expression cassette. The different elements follow the same colour scheme as in Figure 1: blue for the 35S promoter and *nos* terminator, red for the 5S0 5’UTR, green for the cloning site, and yellow for the 3S0 3’UTR. Restriction sites indicated in Figure 1 are underlined in the sequence: Pac1 at the 5’ end, BsmB1 in 5S0, Bsa1 in the cloning site, Sap1 in 3S0, and Asc1 at the 3’ end.
