# Supplementary material for: Improving plant transient expression through the rational design of synthetic 5′ and 3′ untranslated regions
Source: Plant Methods. 2019 Sep 18;15:108. doi: 10.1186/s13007-019-0494-9 (PMC6749642; doi:10.1186/s13007-019-0494-9)
Supplement: Supplementary file 2 — Additional file 2. Sequences of the cloning fragments of the synthetic 5′ and 3′UTRs. Restriction sites (BsmB1 for 5′UTRs and Sap1 for 3′UTRs) are in bold and italicized. The sequence of the UTR once cloned into the expression cassette is underlined. Yellow: simplified synthetic CITE component complementary sequences. Green: synthetic CIRE component sequence. Blue: simplified BTE sequence. Magenta: CPMV RNA-2 3′UTR Y-loop structure. [file 13007_2019_494_MOESM2_ESM.docx]

>5S0

tttaa***gagacg***caaccacaacgctctaacgcaatcaatctacattatattaaa***cgtctc***taaaa

>5S1

***cgtctc***gtttatcaacaacaaccacaacgctctaacgcaatcaatctacattatatcaaacttaaaac***gagacg***

>5S2

***cgtctc***gtttaaccgaagcaaggaccacaactccttgctctaactcaatcaatctacattatattaatcaaacttaaaac***gagacg***

>5S3

***cgtctc***gtttaaacaaacagagcaaggaccacaactccttcgatatacttcttatcggctctaactcaatcaatctacattatattaatcaaacttaaaac***gagacg***

>3S0

cgcc***gaagagc***gcatcggatctaataataaacttaggcaataaatttcgacatcataataaagccacgtatctacatcaacacaagattagtattttcaaaactgttttcagcagaacacatattttcatttttaacgtaattttcatttagcgttagtacagtcaccttcacagaacgtttggagaagtgagg***gctcttc***c

>3S1

***gctcttc***ccgcgcatcggatcactaataataaacttcaggcaataaatttcgacatcataataaagccacgtcatctacatcaacacaagattagtcattttcaaaactgttttcagcagaacacatattttcatttttaataaacgtacattttcatttagcgttagtacagtcaccttcacagaacgtttggagattttagtgagggatt***gaagagc***

>3S2

***gctcttc***ccgcgtaaaataaagccacgtcatctacatcaacacaagattagtcattttgatt***gaagagc***

>3S3

***gctcttc***ccgcgcaataaattttagctacgtatctaataactaagattagcatttctatgtgagcgagttttctgtgctaagtcagtgtttattttgcagagtcgtcccttcagcaaggacacaaaaagattttaattttattgatt***gaagagc***

>3S4

***gctcttc***ccgcgcaataaattcaatcgtcattcaaagttgtggttgattgactgattcctaagattagcatttctatgtgagcgagatttctgtgctaagtcagtgtttatattgtctttgtgagctcctgtttagcaggtcgtcccttcagcaaggacacaaaaagataattttattgatt***gaagagc***

>3S5

***gctcttc***ccgcgcaataaattcagcgttagtacagtcaccttcacagaaccatttaactagagattcctaagattagcatttctatgtgagcgagatttctgtgctaagtcagtgtttatattgtctttgtgagctcctgtttagcaggtcgtcccttcagcaaggacacaaaaagataattttattgatt***gaagagc***

>3S6

***gctcttc***ccgcgcaataaattcaatcgtcattcaaagttgtggttgattgactgattcctaagattagcatttctatgtgagcgagatttctgtgctaagtcagtgtttattagagtaagagagctctcgtataccgagtcgtgccatctgcaagcacactattactttaataattgatt***gaagagc***

>3S7

***gctcttc***ccgcgcaataaattcaggatcctgggaaacaggatgtcggaaagttgtggtttccgattgattcctaagattagcatttctatgtgagcgagatttctgtgctaagtcagtgtttattagagtaagagagctctcgtataccgagtcgtgccatctgcaagcacactattactttaatgatt***gaagagc***

>3S8

***gctcttc***ccgcgcaataaattcaatcgtcattcaaagttgtggttgattgactgattcctaagattagcatttctatgtgagcgagatttctgtgctaagtcagtgtttattagagtaagagagctcctgtataccaggtcgtcccatctgcaaggacactattactttaataattgatt***gaagagc***

**Additional File 2 (.docx).** Sequences of the cloning fragments of the synthetic 5’ and 3’UTRs. Restriction sites (BsmB1 for 5’UTRs and Sap1 for 3’UTRs) are in bold and italicized. The sequence of the UTR once cloned into the expression cassette is underlined. Yellow: simplified synthetic CITE component complementary sequences. Green: synthetic CIRE component sequence. Blue: simplified BTE sequence. Magenta: CPMV RNA-2 3’UTR Y-loop structure.
