# Supplementary material for: Improving plant transient expression through the rational design of synthetic 5′ and 3′ untranslated regions
Source: Plant Methods. 2019 Sep 18;15:108. doi: 10.1186/s13007-019-0494-9 (PMC6749642; doi:10.1186/s13007-019-0494-9)
Supplement: Supplementary file 3 — Additional file 3. Sequence of GFP used in this study. This is a version of eGFP which contains solubility-enhancing point mutations [1, 14]. [file 13007_2019_494_MOESM3_ESM.docx]

>GFP

atggtgagcaagggcgaggagctgttcaccggggtggtgcccatcctggtcgagctggacggcgacgtaaacggccacaagttcagcgtgcgcggcgagggcgagggcgatgccacctacggcaagctgaccctgaagttcatctgcaccaccggcaagctgcccgtgccctggcccaccctcgtgaccaccctgacctacggcgtgcagtgcttcagccgctaccccgaccacatgaagcagcacgacttcttcaagtccgccatgcccgaaggcttcgtccaggagcgcaccatcagcttcaaggacgacggcaactacaagacccgcgccgtggtgaagttcgagggcgacaccctggtgaaccgcatcgagctgaagggcaccgacttcaaggaggacggcaacatcctggggcacaagctggagtacaacttcaacagccacaacgtctatatcaccgccgacaagcagaagaacggcatcaaggccaacttcaccgtgcgccacaacgtggaggacggcagcgtgcagctcgccgaccactaccagcagaacacccccatcggcgacggccccgtgctgctgcccgacaaccactacctgagcacccagacggtgctgagcaaagaccccaacgagaagcgcgatcacatggtcctgcacgagtacgtgaacgccgccgggatcactctcggcatggacgagctgtacaagtag

**Additional File 3 (.docx).** Sequence of GFP used in this study. This is a version of eGFP which contains solubility-enhancing point mutations (Cabantous et al., 2005; Pedelacq et al., 2006).
